# Impacts of Replacing Blended Fish Meal With Diverse Animal and Plant Protein Sources and Their Combinations in the Diets on Growth, Feed Availability, Biochemical Composition, and Blood Chemistry of Rockfish (*Sebastes schlegelii*)

**DOI:** 10.1155/anu/5625045

**Published:** 2025-02-27

**Authors:** Md. Farid Uz Zaman, Sung Hwoan Cho

**Affiliations:** ^1^Department of Convergence Study on the Ocean Science and Technology, National Korea Maritime and Ocean University, Busan 49112, Republic of Korea; ^2^Division of Convergence on Marine Science, National Korea Maritime and Ocean University, Busan 49112, Republic of Korea

**Keywords:** combined protein source, feed availability, fish meal substitution, rockfish (*Sebastes schlegelii*)

## Abstract

The impacts of replacing diverse animal and plant protein sources, and their combination of fish meal (FM) on growth, feed consumption and utilization, biochemical composition, and blood chemistry of juvenile rockfish (*S. schlegelii*) were examined. One thousand and eighty juvenile (mean initial weight of 2.5 g) rockfish were assigned to 27 flow-through tanks with a capacity of 250 L each, with 40 fish per tank. Nine isonitrogenous (52.5%) and isolipidic (12.5%) experimental diets were prepared. The control (Con) diet contained 60% FM. In the Con diet, 25% FM protein was substituted with a single protein source of meat meal (MM), chicken by-product meal (CBM), corn gluten meal (CGM), and corn protein concentrate (CPC), as well as the combined MM and CGM, the combined MM and CPC, the combined CBM and CGM, and the combined CBM and CPC, referred to as the MM, CBM, CGM, CPC, CMCG, CMCP, CCCG, and CCCP diets, respectively. Each formulated feed was given to rockfish in triplicate groups and hand-fed twice a day for 12 weeks. Specific growth rate (SGR) of rockfish fed the Con diet was considerably (*p* < 0.001) greater than that of rockfish fed the MM, CBM, CGM, CPC, CMCP, CCCG, and CCCP diets but not considerably different from that of rockfish fed the CMCG diet. Feed consumption of rockfish was not considerably influenced by dietary treatments. Rockfish fed the Con diet had considerably (*p* < 0.0001 for both) greater feed efficiency and protein efficiency ratio than rockfish fed all other diets, except for the CMCG diet. However, the experimental feeds had no considerable impacts on the biological indices, biochemical composition except for the sum of the *n*−3 highly unsaturated fatty acids, plasma, and serum parameters of rockfish. In summary, the combined MM and CGM can substitute 25% FM protein in the diet of rockfish without retardation in SGR, feed availability, and blood chemistry.

## 1. Introduction

Rockfish (*Sebastes schlegelii*) has become one of the most extensively produced marine finfish species in the Republic of Korea (*hereinafter*, Korea) following olive flounder (*Paralichthys olivaceus*) [1]. This fish has been intensively farmed in Korea during the past several decades because of its rapid growth, high disease resistance, and worldwide market value [2]. The yearly production of this fish species was reported to be 14,418 metric tons (MT) in 2023, ranking second to the overall marine fin fish aquaculture production of 79,651 MT in Korea [1]. In Korea, fish farmers usually choose moist pellets (MPs) (made mostly of raw fish) over formulated feed (FF) in rockfish aquaculture [2, 3]. In 2023, for example, 115,610 MT of MP and 11,598 MT of FF were used for rockfish culture [1]. However, the use of FF is highly recommended for sustainable and environmentally friendly aquaculture worldwide in order to restrict disease transmission, prevent water quality deterioration, and reduce aquaculture operating costs [4, 5]. The diet designed for carnivorous fish cultures typically includes a high proportion of fish meal (FM). Numerous studies have been carried out to date to determine the dietary nutrient requirements [6, 7] and the suitability of diverse animal and plant-derived proteins as alternatives to FM [3, 8–12] for rockfish.

Due to its high protein content, balanced indispensable amino acid (AA) and fatty acid (FA) profiles, nutritional digestibility, minerals, and certain vitamins [13, 14], FM is typically utilized as the primary protein source for commercial aquafeeds, including rockfish [8, 11]. However, because of the depletion of marine fish resources (forage fish stocks) and the rapid increase in demand for aquaculture intensification, the global market price of FM has been steadily rising [15, 16]. Therefore, feed nutritionists are increasingly looking for a substitute for FM that is inexpensive and readily available throughout the year. A number of animal and/or plant-derived protein sources with high amounts of protein, lipid, and certain essential AA (EAA) have been proposed as suitable FM replacements in fish feeds [9, 17, 18]. Aquafeed prices might be decreased by replacing FM with an inexpensive and readily accessible animal or plant protein source [19]. Several studies have been conducted to elucidate the dietary replacement impacts of FM with plant and animal proteins on fish.

Meat meal (MM) is a dry, powdery by-product produced by slaughterhouses or meat processing industries. In general, MM is composed of nonedible parts of rendered animals, such as meat scraps and intestines, after being heated, compressed, and extracted with lipids [20]. Owing to its high protein content, low cost, and wide availability, several investigations have been carried out on the dietary substitution of MM for FM, and those studies have proven that various levels of FM could be successfully substituted with MM in the feeds of rockfish [3, 8], olive flounder [4, 20–22] and barramundi (*Lates calcarifer*) [23]. In our recent study, Lee et al. [3] unveiled that FM up to 80% (44% FM protein) in a 55% FM-based diet could be replaced with MM (crude protein: 68.2% and crude lipid: 12.8%) without adversely influencing the growth and feed utilization of rockfish. However, Lee and Lee [8] suggested that FM up to 29.1% (16% FM protein) in a 55% FM-based diet could be replaceable with MM (crude protein: 74.4% and crude lipid: 10.8%) without compromising the growth performance and feed utilization of rockfish in the 8-week feeding experiment.

Chicken by-product meal (CBM) is an animal-based protein source derived from the clean, dry, ground, and rendered sections of the carcass of slaughtered chickens [15]. Due to its high quantities of crude protein and lipid, CBM is regarded as one of the most affordable, easily accessible, and new sources of feed components that may replace FM in fish feed [15]. The nutritional quality and content of chicken by-products like CBM may vary based on their handling and processing [24]. Although a few studies investigated replacement effect of FM with poultry by-product meal in various fish species [25–28], usage of CBM as a substitute for FM in the diet of rockfish would be unknown. Ha et al. [15] suggested that FM up to 50% could be substituted by CBM in a 65% FM-based diet without adversely affecting the growth and feed utilization of olive flounder.

A significant by-product of plant origin, corn gluten meal (CGM) is primarily produced from the milling of maize. With a high protein content (60%–70% dry matter), low levels of fiber and antinutritional components, and a cheap and reliable supply, CGM is recognized as a cost-effective alternative protein source for FM and is often utilized in aquafeeds [18, 29]. However, the AA profile of CGM is imbalanced, particularly with deficiencies in lysine and arginine [30]. Several studies have tried to determine the effects of switching from FM to CGM on different carnivorous fish, including gilthead sea bream (*Sparus aurata* L.) [31], spotted rose snapper (*Lutjanus guttatus*) [18], cobia (*Rachycentron canadum*) [32], Asian sea bass (*L. calcarifer*) [29], and olive flounder [33]. However, there is a paucity of studies on the use of CGM as a substitute for FM in the rockfish feed.

The plant-derived processed product known as corn protein concentrate (CPC), which is made from corn by enzymatically removing nonprotein components, has a high content of crude protein and carotenoids but a low level of ash and starch, making it an appropriate FM substitute [34, 35]. However, up to date, no available data has been reported on the suitability of the use of CPC as a FM replacer in the diet of rockfish. Several commercially cultured fish including red hybrid tilapia (*Oreochromis* sp.) [35], Nile tilapia (*Oreochromis niloticus* [34], red drum (*Sciaenops ocellatus*), and shortfin corvina (*Cynoscion parvipinnis*) [36] have had portions of FM substituted with CPC in their diets.

Animal and plant protein sources often appear to have certain drawbacks or restrictions as compared to FM. Replacing FM with a single animal or plant protein source may cause several adverse effects on fish, such as deficiency of EAAs and poor digestibility, which often lead to impaired fish growth [37]. Particularly, the EAAs, such as lysine and methionine, are likely to be insufficient if FM is substituted by a single source of animal or plant protein in feeds [38]. According to reports [39, 40], combining protein sources from both plants and animals may result in supplemental AA patterns. Additionally, a number of studies [12, 40–42] have suggested combining different protein sources to obtain a balanced nutritional composition, complement AA profiles, and cover up any unappealing ingredients contained in feed ingredients. It has been revealed in recent studies [37, 43] that FM up to 50% could be substituted with the combination of CGM and MM, and MM and CBM in the diets of rockfish and red sea bream (*Pagrus major*), respectively. Although numerous studies on dietary effects of using single animal and plant protein source including MM, CBM, CGM, and CPC as the alternatives for FM have been performed in various fish species, no study on the use of respective animal and plant protein source or their combination as the alternative to FM in the rockfish diets has been known yet.

The purpose of this study, thus, is to evaluate the dietary replacement effects of various animal and plant protein sources, and their combinations for FM on growth, feed availability, biochemical composition, and blood chemistry of rockfish.

## 2. Materials and Methods

### 2.1. Experimental Conditions

Juvenile rockfish were bought from a commercial hatchery (Hyun Fisheries, Buan-gun, Jeollabuk-do, Korea) and underwent a 10-day acclimatization period before the outset of the feeding experiment. Throughout the acclimatization phase, fish were supplied with commercial feed (54% crude protein and 8% crude lipid; National Federation of Fisheries Cooperatives Feed, Uiryeong-gun, Gyeongsangnam-do, Korea) twice daily. A total of 1,080 juvenile (initial weight of 2.5 g) fish were uniformly dispersed into 27 flow-through tanks with a capacity of 250 L each, with 40 fish per tank. Each tank was equipped with a proper aeration system throughout the experimental period and continuously received a mixture of underground seawater and sand-filtered seawater ( = 1:1) at flow rate of 4.2 L/min. The water quality was tracked on a daily basis over the experimental period using the AZ-8603 multiparameter water quality instrument (AZ Instrument, Taichung, Taiwan). The parameters assessed were as follows: water temperature varied from 15.7 to 24.1°C (20.5 ± 0.28°C; mean ± SD), dissolved oxygen varied from 7.0 to 8.7 mg/L (7.5 ± 0.04 mg/L), salinity varied from 31.4 to 33.6 g/L (32.6 ± 0.06 g/L), and pH varied from 6.8 to 7.7 (7.2 ± 0.02). All experimental diets were administered to rockfish in triplicate groups and carefully hand-fed twice daily (08:00 and 17:00) to achieve apparent satiation for a period of 12 weeks. Siphon cleaning was performed daily to avoid the risk of deterioration of the water quality, and dead fish were promptly removed upon observation.

### 2.2. Experimental Feeds

Nine experimental feeds were formulated (Table 1). The control (Con) diet contained 60% FM (blended sardine meal, pollock meal, and tuna by-product meal at a ratio of 1:1:1) and 8% soybean meal as the predominant protein sources. The Con diet had 5.5% fish oil and 17.2% wheat flour for the lipid and carbohydrate sources, respectively. In the Con diet, 25% FM protein, being equivalent to 41.7% of FM, was substituted with the single protein source of MM, CBM, CGM, and CPC, and the combined MM and CGM, the combined MM and CPC, the combined chicken by-product meal and CGM, and the combined chicken by-product meal and CPC, referred to as the MM, CBM, CGM, CPC, CMCG, CMCP, CCCG, and CCCP diets, respectively. Each of the experimental diets was prepared to be isonitrogenous at 52.0% and isolipidic at 12.5%. A laboratory pellet extruder (Dongsung Mechanics, Busan, Korea) was used to pelletize the contents of the experimental feeds after thoroughly mixing them with water at a 3:1 ratio to create a uniform mixture and then air-dried at 40°C for 48 h. Before usage, the experimental feeds were kept at –20°C. According to Cho et al. [44] and Kim et al. [45]'s studies, the experimental feeds were prepared to fulfill the nutritional needs for the growth of rockfish.

### 2.3. Measurements of the Biological Indices of Rockfish

At the termination of the 12-week feeding experiment, all live fish in each tank underwent a 24-h period of fasting, followed by anesthesia using tricaine methanesulfonate (MS-222) at a concentration of 100 ppm. The fish in each tank were then collectively counted and weighed to determine survival rates and weight gain. In each tank, 10 randomly selected anesthetized fish were chosen for the calculation of the viscerosomatic index (VSI) and hepatosomatic index (HSI). The biological measurements were calculated using the following formulas: specific growth rate (SGR, %/day) = [Ln final body weight of fish (g)−Ln initial body weight of fish (g)] × 100/days of feeding (84 days), feed efficiency (FE)  = weight gain of fish/feed consumption, protein efficiency ratio (PER)  = weight gain of fish/protein consumption, protein retention (PR, %)  = protein gain of fish × 100/protein consumption, condition factor (CF, g/cm^3^)  = fish weight (g) × 100/total length (cm)^3^, VSI (%)  = viscera weight × 100/fish weight, and HSI (%)  = liver weight × 100/fish weight.

### 2.4. Analysis of the Biochemical Composition of the Experimental Diets and Fish

The experimental diets, 10 rockfish prior to the feeding experiment, and remaining rockfish (≥20) from each tank at the end of the 12-week feeding trial except for 10 fish for the blood chemistry analysis were homogenized and used for the biochemical composition analysis. Following the AOAC standard method [46], the chemical composition of the experimental feeds and whole body of rockfish were analyzed. The Kjeldahl method, facilitated by the Kjeltec 2100, Distillation Unit from Foss Tecator (Hillerod, Denmark), was employed to ascertain the crude protein content. The crude lipid content was evaluated through an ether-extraction method, employing the Soxtec TM 2043 Fat Extraction System, manufactured by Foss Tecator (Hillerod, Denmark). For moisture content analysis, the samples underwent desiccation in an oven maintained at 105°C for a period of 24 for a duration of 24 h. The ash content was measured by subjecting the samples to a muffle furnace at 550°C for 4 h.

By hydrolyzing the meals with 6 N HCl for 24 h at 110°C, followed by ion exchange chromatography using an AA analyzer (L-8800 Auto-analyzer: Hitachi, Tokyo, Japan), all AA (apart from methionine and cysteine) of the experimental diets and whole body of fish were examined. The materials were oxidized with performic acid at a temperature below 5°C for 24 h to produce methionine sulfone and cysteic acid, and they were then twice freeze-dried with deionized water for the measurement of methionine and cysteine. The technique utilized for the other AAs was followed when hydrolyzing and analyzing the freeze-dried samples.

FA from the experimental diets and whole-body fish were extracted by a mixture of chloroform and methanol (2:1 v/v) based on the method of Folch, Lees, and Stanley [47], and FA methyl esters were prepared by transesterification with 14% BF3-MeOH (Sigma, St. Louis, MO, USA). FA methylesters were analyzed using gas chromatography (Trace GC, Thermo, Waltham, MA, USA) equipped with a flame ionization detector and a SPTM-2560 capillary column (100 m × 0.25 mm I.d. film thickness 0.20 µm; manufactured by Supelco, Bellefonte, PA, USA).

### 2.5. Blood Chemistry of Fish

For the purpose of analyzing the plasma and serum chemistry of rockfish, blood samples were obtained via five heparinized syringes (1 mL) and five nonheparinized syringes from the caudal vein of 10 anesthetized rockfish per tank, respectively. Following centrifugation (2,716 × *g*, 10 min) at 4°C, serum and plasma were obtained and kept at −70°C in a refrigerator until analysis. Plasma was utilized to determine the aspartate aminotransferase (AST), alanine aminotransferase (ALT), alkaline phosphatase (ALP), total bilirubin (T-BIL), total cholesterol (T-CHO), triglyceride (TG), total protein (TP), and albumin (ALB), analyzed by the kits (Fuji-Chem NX500i, Fujifilm, Tokyo, Japan). The superoxide dismutase (SOD) and lysozyme activity were analyzed using serum. The SOD was measured by ELISA kit (MyBioSource, cat. No. MBS705758) according to the manufacturer's instructions. A microplate reader (Tecan Infinite 200 PRO, Zürich, Switzerland) was used to detect absorbance, and standard curves were plotted to calculate concentration. The turbidimetric assay for lysozyme was done based on Lange, Gudmundsdottir, and Magnadottir [48]'s study. Lysozyme analysis was carried out according to the same procedures and methods in Lee et al. [3]'s study.

### 2.6. Statistical Analysis

Using SPSS version 24.0 (SPSS Inc., Chicago, IL, USA), the data were submitted to one-way analysis of variance (ANOVA), and Tukey's honestly significant difference (HSD) post hoc test was performed to assess the significant differences among the means of treatments. The normality and homogeneity of treatment variances in all data were assessed by Shapiro–Wilk's test and Levene's test, respectively. Prior to the statistical analysis, all percentage values were converted using an arcsine function.

## 3. Results

### 3.1. AA Profiles of the Experimental Feeds

The AA profiles of several protein sources (FM, MM, CBM, CGM, and CPC) and the experimental diets are shown in Table 2. Leucine, and glutamic and aspartic acids were the most abundant AA among the EAA and nonessential AA (NEAA), respectively, in the main protein sources and the experimental feeds. However, it was found that all protein sources and the experimental feeds had somewhat reduced levels of isoleucine, lysine, methionine, threonine, tryptophan, and valine among the EAA and aspartic acid among the NEAA compared to FM and the Con diet, respectively.

### 3.2. FA Profiles of the Experimental Feeds

FA profiles of the various protein sources and the experimental diets are presented in Table 3. Total saturated FA (∑SFA) and monounsaturated FA (∑MUFA) in animal protein sources including MM and CBM were higher and lower than those in FM, respectively, but all lower for plant protein sources including CGM and CPC. Both CGM and CPC had an extremely high linoleic acid (C18:2*n*−6) (54.09% and 54.22% of total FA, respectively). Total *n*−3 highly unsaturated FA (∑ *n*−3 HUFA) including eicosapentaenoic acid (EPA, 20:5*n*−3) and docosahexaenoic acid (DHA, 22:6*n*−3) in the Con diet was relatively high over those in all other experimental feeds.

### 3.3. Survival and Growth Performance of Rockfish

The survival of rockfish was not considerably (*p* > 0.05) influenced by dietary treatments (Table 4). However, the weight gain of rockfish fed the Con diet was considerably (*p* < 0.0001) higher than that of rockfish fed all other diets. The SGR of rockfish fed the Con diet was considerably (*p* < 0.001) greater than that of rockfish fed the MM, CBM, CGM, CPC, CMCP, CCCG, and CCCP diets, but not considerably (*p* > 0.05) different from that of rockfish fed the CMCG diet. However, no considerable (*p* > 0.05) difference in SGR was observed among rockfish fed all experimental diets, except for the Con diet.

### 3.4. Feed Availability and Biological Measurements of Rockfish

No considerable difference (*p* > 0.07) in feed consumption was observed among rockfish fed all experimental diets (Table 5). The FE and PER of rockfish fed the Con diet were considerably higher (*p* < 0.0001 for both) than that of rockfish fed all other diets, except for the CMCG diet, where the difference was not considerable (*p* > 0.05). Regarding PR, rockfish fed the Con diet had considerably (*p* < 0.0001) higher values compared to rockfish fed all other diets, except for the MM and CMCG diets. The CF values ranged from 1.71 to 1.84 g/cm^3^, the VSI values ranged from 9.60% to 10.91%, and the HSI values ranged from 2.96% to 3.56%. However, the experimental diets did not show any considerable (*p* > 0.5, *p* > 0.5, and *p* > 0.4, respectively) impact on these values.

### 3.5. Chemical Composition of the Whole-Body Rockfish

The moisture content of the whole-body rockfish varied from 69.8% to 74.5%, while the crude protein content varied from 14.2% to 16.1% (Table 6). The crude lipid content varied from 5.9% to 8.4% and the ash content varied from 3.9% to 4.9%. However, the experimental feeds did not show any considerable (*p* > 0.05 for all) impact on the proximate compositions of the whole-body rockfish.

### 3.6. Blood Chemistry of Rockfish

The plasma AST of rockfish varied from 106.0 to 156.7 IU/L, ALT varied from 13.7 to 25.0 IU/L, ALP varied from 212.3 to 526.3 IU/L, T-BIL varied from 1.7 to 3.0 mg/dL, T-CHO varied from 211.3 to 251.7 mg/dL, TG varied from 309.3 to 472.0 mg/dL, TP varied from 4.8 to 6.0 g/dL, and ALB varied from 1.1 to 2.1 g/dL (Table 7). None of the hematological measurements of rockfish was considerably (*p* > 0.05 for all) altered by dietary treatments.

Lysozyme activity of rockfish changed from 746.8 to 756.6 U/mL, and SOD changed from 3.7 to 4.1 ng/mL (Table 7). These values were not considerably (*p* > 0.9 and *p* > 0.6) altered by dietary treatments.

### 3.7. AA Profiles of the Whole-Body Rockfish

None of the AA profiles of the whole-body rockfish was considerably (*p* > 0.05) affected by dietary treatments (Table 8).

### 3.8. FA Composition of the Whole-Body Rockfish

The FA profiles of the whole-body rockfish fed the experimental diets for a period of 12 weeks are exhibited in Table 9. With the exception of ∑*n*−3 HUFA, all FA profiles of the whole-body rockfish were not considerably influenced (*p* > 0.05) by dietary treatments. The ∑*n*−3 HUFA in the whole-body rockfish fed the Con diet was considerably (*p* < 0.02) higher than that of the fish fed all other diets. However, no considerable (*p* > 0.05) differences were observed in the ∑*n*−3 HUFA among rockfish fed the MM, CBM, CGM, CPC, CMCG, CMCP, CCCG, and CCCP diets.

## 4. Discussion

The SGR values (2.65%–3.20%/day) of rockfish achieved in this study were superior or comparable to values reported in the same fish species with similar size in other studies (2.64%–2.93%/day, 1.89%–2.40%/day, and 1.97%–3.23%/day for initial weight of 2.3, 3.2, and 2.5 g, respectively) [3, 9, 52], proving that growth performance of rockfish was well attained. Although the weight gain of rockfish fed the Con diet was superior to fish fed all other diets, no statistical differences in SGR and feed consumption of rockfish fed the Con and CMCG diets would be found in the 12-week feeding experiment in this study. This might indicate that 25% FM protein, being equivalent to 41.7% of FM, can be substitutable with the combined MM and CGM without compromising SGR and feed consumption of rockfish.

However, the MM and CGM diets led to inferior growth performance to rockfish fed the Con diet. Likewise, Lee and Lee [8] revealed that FM up to 29.1% could be successfully replaced with MM in rockfish feeds when rockfish were fed with a 55% FM-based diet or diets replacing 14.5%, 29.1%, 43.6%, and 58.2% FM with MM. Lee, Yoo, and Lee [53] also unveiled that FM up to 18% could be substitutable with CGM without adversely affecting weight gain of rockfish when 9%, 18%, and 27% FM were substituted with CGM in the 55% FM-based diet. In considering the results of Lee and Lee [8], Lee, Yoo, and Lee [53], and this study, the substitutability of the combined protein source of MM and CGM in this study could be improved for higher levels of FM substitution (41.7% of FM) compared to the single protein sources of MM (29.1%) or CGM (18%) reported in previous studies. Similarly, several studies [12, 40, 42] also emphasized that using multiple protein sources instead of a single protein source allows for higher levels of FM substitution in diets without compromising fish growth and FE. In particular, Gunathilaka et al. [43] also suggested that the combined MM and CBM were efficient in replacing 50% FM in the feeds of red sea bream. A most recent study by Lee et al. [37] also reported that a combination of CGM and MM could replace FM up to 50% in the rockfish diet without compromising growth performance.

In the context of using economical protein sources with potentially lower AA content compared to FM, it becomes important to consider satisfying the EAA requirements of fish by utilizing protein ingredients with complementary AA profiles [54]. Among the EAAs, lysine and methionine are considered crucial AAs, which are often the limiting AAs in commercial feed [55, 56]. The lysine contents of the MM, CBM, and CMCG diets in this study were 3.29%, 3.13%, and 2.90%, respectively, which are comparable to the lysine content in the Con diet (3.45%) and meet the lysine requirement (2.99%) for rockfish as reported by Yan et al. [49]. This could be a reason why the CMCG diet led to a comparable SGR of rockfish fed the Con diet in this study. However, the methionine content in all experimental diets including the Con diet seemed slightly lower based on Yan et al. [50]'s study, which determined dietary methionine requirement of 1.37% in the presence of 0.12% cysteine for rockfish. Interestingly, the cysteine content of all experimental feeds varied from 0.61% to 0.90% in this study, which was higher than the cysteine level (0.12%) in diets as reported by Yan et al. [50]. This higher cysteine content might potentially reduce the dietary methionine requirement of rockfish in this study. It has been shown in previous studies that cysteine is capable of sparing 40% and 50% of the dietary methionine requirements of stinging catfish (*Heteropneustes fossilis*) and red drum, respectively [57, 58].

In the diets of most marine fish, certain levels of *n−*3 HUFA including EPA and DHA are necessary to achieve normal growth and health [59–62]. EPA and DHA play vital roles in performing various physiological functions in the fish body, such as maintenance of cellular membrane integrity, regulation of immune and inflammatory responses, and carbohydrate and lipid metabolism [63, 64]. Generally speaking, most marine fish possess a limited or nonexistent ability to synthesize EPA, DHA, and arachidonic acid (ARA, 20:4*n*−6) at levels that meet their physiological needs, making these FA indispensable and requiring dietary sources to fulfill their nutritional requirements [63, 65]. Although some studies had revealed dietary ∑*n*−3 HUFA requirements for several finfish [66], there is still a paucity of information on requirements of these respective EPA, DHA and ARA. The dietary requirement for ∑*n*−3 HUFA in juvenile rockfish has been estimated to be 0.9% of the diet [51], which is equivalent to 7.25% of the total FA. Among the experimental diets, only the Con diet (8.59% of total FA) appeared to satisfy this requirement. However, all other experimental diets, except the Con diet, showed a slightly lower ∑*n*−3 HUFA compared to the dietary required level [51]. This discrepancy in ∑*n*−3 HUFA could explain why rockfish fed the Con diet exhibited a higher weight gain compared to rockfish fed all other diets.

No distinct alteration was observed in feed consumption among the experimental diets, but comparable FE, PER, and PR were noticed in rockfish fed the CMCG diet compared to the Con diet in this study. This indicates that substituting 25% FM protein with the combined MM and CGM did not influence the acceptability of the feed by rockfish, but it did significantly impact feed utilization. However, all experimental diets except for the CMCG diet achieved inferior FE and PER compared to the Con diet. Similar findings have been reported in other studies. Jeon et al. [9] found that replacing various levels (10%, 20%, 30%, 40%, 60%, 80% and 100%) of FM with TBM did not influence feed consumption, but had a marked effect on feed utilization (PER and PR) in rockfish. Kader et al. [41] also found no outstanding difference in feed consumption, but distinct alterations in growth performance and feed utilization of olive flounder when FM was substituted with fermented SBM and squid by-product blend in diets. Various alternative animal protein sources for FM also did not alter feed consumption but noticeably affected the growth and feed utilization of olive flounder [4, 15]. However, a recent study by Jeong and Cho [67] demonstrated that up to 50% of FM could be substituted by dietary alternatives, including CBM, TBM, and MM, with the inclusion of jack mackerel meal as a feed enhancer, which had a significant influence on feed consumption but no influence on feed utilization in olive flounder. Regarding the biological indices (CF, VSI, and HSI), no marked differences were noticed in rockfish fed the experimental feeds for 12 weeks in this study. This is consistent with a study by Choi et al. [68], where 20% of FM was replaced by combined animal and plant protein sources without any alteration in the CF, VSI, and HSI of olive flounder. Similar findings have also been reported in other studies involving rockfish [8, 69], olive flounder [15, 67], and humpback grouper (*Cromileptes altivelis*) [70].

No remarkable alterations were found in the chemical composition of the whole-body rockfish in this study, indicating that replacing 25% FM protein with MM, CBM, CGM, and CPC or their combination had no impact on the nutritional changes of rockfish. These findings are consistent with earlier studies [3, 4, 15, 20, 43, 71] demonstrating that dietary replacement of various animal and plant protein sources for FM did not change the proximate composition of the whole-body olive flounder, rockfish, and red sea bream. Contrary to this study, however, several contradictory findings demonstrated that dietary FM replacement by various alternatives (animal and/or plant protein sources) affected on the chemical composition of the whole-body rockfish [37], olive flounder [21], and spotted rose snapper [18].

Plasma biochemical measurements are commonly considered as a useful tool for clinical diagnosis in fish, providing valuable insights into their nutritional and physical conditions [72, 73]. In this study, the plasma measurements of rockfish were not altered by the experimental feeds, being consistent with findings in rockfish-fed diets with varying levels of TBM as a FM substitute [9, 74]. Likewise, dietary substitution of FM with various alternative proteins did not induce changes in the blood chemistry of olive flounder [4, 15, 22], and rockfish [69]. Additionally, Lee and Lee [8] found no effect on blood chemistry measurements, such as TP and total glucose, when replacing FM with MM in the feeds of rockfish. However, in contrast to the present study, various alternative protein sources used for FM replacement significantly influenced the plasma constituents in olive flounder diets [33, 41, 75].

No remarkable differences in lysozyme activity and SOD of rockfish in this study imply that replacing 25% FM protein with various animal and plant and their combined ingredients in the feeds of rockfish did not compromise the lysozyme activity and SOD. Similar findings were observed in olive flounder in Ha et al. [15, 20]'s studies, where substituted diets by MM and CBM as the FM alternatives showed no distinctive differences in the lysozyme activity and SOD. Dossou et al. [76] also demonstrated that substituting FM with yeast-fermented rapeseed meal in the diet of red sea bream did not result in differences in lysozyme and total peroxidase activities. However, in contrast to these studies, Khosravi et al. [77] found that dietary inclusion of shrimp, tilapia, or krill hydrolysate in low FM diets significantly altered the lysozyme activity and SOD of red sea bream.

The experimental feeds used in this experiment did not change the AA profiles of the whole body of rockfish, being consistent with the findings by Jeon et al. [9], and Li and Cho [74], where substituting FM with tuna by-product meal did not change the AA profiles of rockfish. Likewise, Ha et al. [15] revealed that incorporating CGM as an FM substitute had no significant impact on the AA profiles of olive flounder. Furthermore, Kim et al. [38] observed that the AA profiles of the whole-body olive flounder remained unaffected when up to 30% of FM was replaced with TBM. In contrast to these findings, however, Deng et al. [78] demonstrated that replacing FM with SPC in the feeds of olive flounder had a considerable impact on the whole-body AA profiles.

The higher ∑*n−*3 HUFA in the whole-body rockfish fed the Con diet compared to fish fed all other feeds were well responded from the relatively higher ∑*n*−3 HUFA in the Con diet used in this experiment. This observation aligns with the findings of Lazo et al. [62], who noted that the FA profiles of diets are often reflected in the FA composition of fish's whole body. Similarly, the substitution of FM by various proteins has been shown to alter the FA profiles of the whole-body fish [20, 62, 79]. However, in contrast to these findings, Lee et al. [3] demonstrated that the FA profiles of the whole-body rockfish remained unaffected when different levels of FM were substituted with MM in diets.

Replacing FM with animal and plant protein sources in aquafeeds is considered an environmentally sustainable approach, as it reduces reliance on limited marine fish resources, specifically forage fish stocks commonly used for FM production [13, 80, 81]. The findings of this study demonstrate that the combined MM and CGM can effectively replace 25% FM protein in the rockfish diets without adversely impacting SGR, feed consumption, feed utilization (FE, PER, and PR), and blood chemistry. This result has significant practical implications for the aquaculture industry, particularly for rockfish farm. By utilizing the combined MM and CGM, aquaculture industry can potentially reduce the reliance on FM. This allows feed producers to lower production cost while maintaining the growth performance of fish and contributing to more sustainable and economically viable aquaculture practices.

In conclusion, rockfish fed the CMCG diet exhibited comparable SGR, feed consumption, and feed utilization (FE, PER, and PR) to rockfish fed the Con diet. However, no statistical differences in feed consumption, biological indices, and biochemical composition except for the ∑*n*−3 HUFA content, and blood chemistry of rockfish fed all experimental feeds were found. Therefore, the combined MM and CGM may substitute 25% FM protein in the diet of rockfish without retardation in SGR, feed availability, biochemical composition except for the ∑*n*−3 HUFA content, and plasma and serum parameters.

## Figures and Tables

**Table 1:** Ingredients and chemical composition of the experimental diets (%, DM basis).

	Experimental diets								
	Con	MM	CBM	CGM	CPC	CMCG	CMCP	CCCG	CCCP
Ingredients (%)									
Fish meal^a^	60.0	35.0	35.0	35.0	35.0	35.0	35.0	35.0	35.0
Meat meal^b^	—	19.5	—	—	—	9.8	9.8	—	—
Chicken by-product meal^c^	—	—	27.1	—	—	—	—	13.6	13.6
Corn gluten meal^d^	—	—	—	26.3	—	13.2	—	13.2	—
Corn protein concentrate^e^	—	—	—	—	22.1	—	11.0	—	11.0
Soybean meal^f^	8.0	8.0	8.0	8.0	8.0	8.0	8.0	8.0	8.0
Squid liver powder^g^	3.0	3.0	3.0	3.0	3.0	3.0	3.0	3.0	3.0
Poultry by-product meal^h^	3.0	3.0	3.0	3.0	3.0	3.0	3.0	3.0	3.0
Krill meal	0.5	0.5	0.5	0.5	0.5	0.5	0.5	0.5	0.5
Wheat flour	17.2	22.3	16.5	13.6	18.7	17.8	20.5	15.0	17.6
Fish oil	5.5	5.9	4.1	7.8	6.9	6.9	6.4	5.9	5.5
Lecithin	1.0	1.0	1.0	1.0	1.0	1.0	1.0	1.0	1.0
Mono calcium phosphate	0.2	0.2	0.2	0.2	0.2	0.2	0.2	0.2	0.2
Mineral premix^i^	0.5	0.5	0.5	0.5	0.5	0.5	0.5	0.5	0.5
Vitamin premix^j^	0.5	0.5	0.5	0.5	0.5	0.5	0.5	0.5	0.5
Vitamin C	0.5	0.5	0.5	0.5	0.5	0.5	0.5	0.5	0.5
Choline	0.1	0.1	0.1	0.1	0.1	0.1	0.1	0.1	0.1
Nutrients (%)									
Dry matter	97.8	98.0	97.6	97.4	97.1	97.5	97.6	97.3	97.4
Crude protein	51.9	52.9	52.3	52.9	52.1	52.2	52.3	52.8	52.3
Crude lipid	12.4	12.5	12.7	12.5	12.5	12.5	12.5	12.5	12.5
Ash	12.3	9.7	12.2	9.4	8.8	9.3	9.3	10.9	10.7

Abbreviations: CBM, chicken by-product meal; CGM, corn gluten meal; CPC, corn protein concentrate; MM, meat meal.

^a^Fish meal (FM) (crude protein: 70.0%, crude lipid: 9.0%, ash: 16.2%) are composed of sardine meal, pollock meal, and tuna by-product meal at a ratio of 1:1:1. Sardine meal and pollock meal were imported from Chile and tuna by-product meal was purchased from Woojinfeed Ind. Co. Ltd. (Incheon Metropolitan City, Korea).

^b^Meat meal (MM) (crude protein: 85.6%, crude lipid: 8.5%, ash: 5.9%) was purchased from Hwasong Ind. Co. Ltd. (Seogwipo-si, Jeju Special Self-Governing Province, Korea).

^c^Chicken by-product meal (CBM) (crude protein: 65.0%, crude lipid: 13.6%, ash: 16.4%) was purchased from Hwasong Ind. Co. Ltd. (Seogwipo-si, Jeju Special Self-Governing Province, Korea).

^d^Corn gluten meal (CGM) (crude protein: 68.7%, crude lipid: 0.2%, ash: 1.6%) was purchased from Hyunjin Provender Co. Ltd. (Icheon-si, Gyeonggi-do, Korea).

^e^Corn protein concentrate (CPC) (crude protein: 78.3%, crude lipid: 3.7%, ash: 1.0%) was imported from Cargill (Minneapolis, MN, USA).

^f^Soybean meal (crude protein: 52.6%, crude lipid: 2.1%, ash: 7.2%).

^g^Squid liver powder (crude protein: 47.5%, crude lipid: 19.7%, ash: 7.2%).

^h^Poultry by-product meal (crude protein: 64.9%, crude lipid: 11.6%, ash: 18.9%).

^i^Mineral premix contained the following ingredients (g/kg mix): MgSO_4_·7H_2_O, 80.0; NaH_2_PO_4_·2H_2_O, 370.0; KCl, 130.0; ferric citrate, 40.0; ZnSO_4_·7H_2_O, 20.0; Ca-lactate, 356.5; CuCl, 0.2; AlCl_3_·6H_2_O, 0.15; KI, 0.15; Na_2_Se_2_O_3_, 0.01; MnSO_4_·H_2_O, 2.0; CoCl_2_·6H_2_O, 1.0.

^j^Vitamin premix contained the following amounts, which were diluted in cellulose (g/kg mix): L-ascorbic acid, 121.2; DL-*α*-tocopheryl acetate, 18.8; thiamin hydrochloride, 2.7; riboflavin, 9.1; pyridoxine hydrochloride, 1.8; niacin, 36.4; Ca-D-pantothenate, 12.7; myo-inositol, 181.8; D-biotin, 0.27; folic acid, 0.68; p-aminobenzoic acid, 18.2; menadione, 1.8; retinyl acetate, 0.73; cholecalciferol, 0.003; cyanocobalamin, 0.003.

**Table 2:** Amino acid profiles (% of the diet) of the various protein sources and experimental diets.

	Protein sources					Requirement	Experimental diets								
	FM	MM	CBM	CGM	CPC		Con	MM	CBM	CGM	CPC	CMCG	CMCP	CCCG	CCCP
Essential amino acids (EAA, %)															
Arginine	3.90	5.71	4.14	2.15	1.99	—	2.89	3.05	3.01	2.52	2.46	2.97	2.90	2.72	2.67
Histidine	1.51	1.61	1.29	1.32	1.35	—	1.15	1.20	1.13	1.14	1.14	1.16	1.15	1.12	1.11
Isoleucine	2.74	1.99	1.95	2.44	2.07	—	2.01	1.80	1.93	1.99	1.93	1.72	1.64	1.71	1.45
Leucine	4.83	4.42	4.08	10.95	12.70	—	3.63	3.46	3.24	5.38	5.40	4.78	4.07	4.29	4.30
Lysine	5.23	4.13	3.92	1.22	0.93	2.99^a^	3.45	3.29	3.13	2.50	2.41	2.90	2.71	2.74	2.69
Methionine	2.17	1.27	1.27	1.75	1.80	1.37^b^	1.23	1.11	1.14	1.25	1.25	1.15	1.20	1.16	1.22
Phenylalanine	2.65	2.50	2.22	4.02	4.48	—	2.05	1.90	1.97	2.47	2.45	2.14	2.31	2.14	2.34
Threonine	2.59	2.42	2.43	2.28	2.44	—	2.02	1.93	1.98	1.83	1.97	1.92	1.94	1.90	1.96
Tryptophan	0.49	0.38	0.47	0.28	0.29	—	0.42	0.38	0.40	0.34	0.36	0.33	0.36	0.37	0.35
Valine	3.38	2.99	2.58	2.92	2.59	—	2.49	2.48	2.42	2.40	2.38	2.15	2.06	2.15	1.94
∑EAA^c^	29.49	27.42	24.35	29.33	30.64	—	21.34	20.6	20.35	21.82	21.75	21.22	20.34	20.30	20.03
Non-essential amino acids (NEAA, %)															
Alanine	4.30	5.46	4.31	4.67	5.67	—	3.06	3.62	3.18	3.28	3.63	3.45	3.52	3.39	3.41
Aspartic acid	5.98	5.57	4.87	3.22	3.74	—	4.39	4.24	4.11	3.94	3.91	4.35	4.07	3.91	3.98
Cysteine	0.94	0.61	0.75	1.30	1.70	0.12^b^	0.66	0.61	0.65	0.86	0.90	0.72	0.75	0.75	0.81
Glutamic acid	8.73	9.73	8.22	11.36	13.80	—	7.12	7.09	7.12	8.69	8.85	8.32	8.42	7.84	7.95
Glycine	4.70	11.94	6.05	1.43	1.68	—	3.36	4.64	4.01	2.70	2.71	3.56	3.61	3.13	3.52
Proline	3.00	7.38	3.94	5.14	6.47	—	2.42	3.45	2.91	3.37	3.22	3.40	3.42	3.31	3.27
Serine	2.22	2.90	2.46	2.72	3.26	—	1.97	1.92	1.84	2.36	2.41	2.25	2.27	2.39	2.40
Tyrosine	1.31	1.44	1.44	2.31	2.84	—	1.02	0.93	0.98	1.49	1.58	1.35	1.37	1.52	1.61
∑NEAA^d^	31.18	46.03	32.04	32.15	39.16	—	24.00	26.50	24.80	26.69	27.21	27.40	27.43	26.24	26.95

Abbreviations: CBM, chicken by-product meal; CGM, corn gluten meal; CPC, corn protein concentrate; MM, meat meal.

^a^Data were obtained from Yan et al. [49]'s study.

^b^Data were obtained from Yan et al. [50]'s study.

^c^∑EAA: total essential amino acids.

^d^∑NEAA: total non-essential amino acids.

**Table 3:** Fatty acid profiles (% of total fatty acids) of the various protein sources and experimental diets.

	Protein sources						Experimental diets								
	FM	MM	CBM	CGM	CPC	Requirement	Con	MM	CBM	CGM	CPC	CMCG	CMCP	CCCG	CCCP
C12:0	0.06	0.16	0.08	0.05	0.04	—	0.06	0.06	0.06	0.04	0.04	0.05	0.05	0.05	0.05
C14:0	5.78	2.43	0.97	0.08	0.10	—	2.40	2.11	1.93	1.65	1.69	1.82	1.84	1.77	1.79
C16:0	21.76	28.08	28.12	13.41	13.37	—	20.85	20.79	22.74	18.85	18.51	19.32	19.72	20.34	20.99
C18:0	4.44	14.99	8.71	2.13	1.88	—	7.68	8.30	8.12	6.76	6.76	7.43	7.21	7.15	7.11
C20:0	0.15	0.20	0.12	0.49	0.44	—	0.23	0.24	0.21	0.26	0.25	0.24	0.24	0.23	0.25
C24:0	1.76	0.03	0.04	0.51	0.51	—	0.60	0.10	0.39	0.40	0.42	0.42	0.41	0.41	0.39
∑SFA^a^	33.95	45.89	38.04	16.67	16.34	—	31.82	31.6	33.45	27.96	27.67	29.28	29.47	29.95	30.58
C14:1*n*−5	0.14	0.05	0.24	0.00	0.00	—	0.07	0.06	0.08	0.05	0.05	0.05	0.05	0.06	0.07
C15:1*n*−5	0.07	0.06	0.27	0.06	0.01	—	0.04	0.04	0.03	0.03	0.03	0.04	0.03	0.03	0.03
C16:1*n*−7	7.06	3.05	5.78	0.18	0.22	—	3.40	2.84	3.45	2.49	2.51	2.69	2.66	2.95	3.09
C17:1*n*−7	0.54	0.45	0.19	0.05	0.03	—	0.35	0.27	0.25	0.22	0.23	0.25	0.27	0.24	0.24
C18:1*n*−9	19.37	45.46	48.02	25.28	25.50	—	24.98	30.10	30.22	25.50	25.75	27.13	27.02	28.35	28.31
C20:1*n*−9	1.57	1.36	1.05	0.36	0.31	—	1.24	1.08	1.07	0.96	0.96	1.02	1.04	1.00	1.01
C22:1*n*−9	3.30	0.03	0.06	0.02	0.02	—	1.59	1.15	1.14	1.06	1.09	1.07	1.14	1.05	1.12
C24:1*n*−9	0.15	0.00	0.00	0.00	0.00	—	0.11	0.12	0.09	0.14	0.14	0.11	0.12	0.12	0.12
∑MUFA^b^	32.20	50.46	55.61	25.95	26.09	—	31.78	35.66	36.33	30.45	30.76	32.36	32.33	33.80	33.99
C18:2*n*−6	2.67	2.30	4.86	54.09	54.22	—	22.87	24.47	20.73	30.84	30.79	28.26	27.95	26.46	25.48
C18:3*n*−3	0.61	0.03	0.06	2.30	2.18	—	3.19	2.31	2.39	3.48	3.35	3.35	3.14	2.98	2.82
C18:3*n*−6	0.17	0.04	0.02	0.04	0.00	—	0.07	0.06	0.05	0.05	0.05	0.05	0.05	0.05	0.05
C20:2*n*−6	0.17	0.09	0.08	0.07	0.05	—	0.14	0.16	0.15	0.16	0.16	0.16	0.16	0.15	0.14
C20:3*n*−3	0.10	0.00	0.00	0.00	0.00	—	0.05	0.05	0.04	0.05	0.05	0.05	0.05	0.05	0.05
C20:3*n*−6	0.09	0.00	0.00	0.00	0.00	—	0.09	0.08	0.08	0.09	0.08	0.08	0.08	0.09	0.09
C20:4*n*−6	0.93	0.15	0.36	0.00	0.01	—	0.43	0.26	0.37	0.30	0.31	0.32	0.33	0.34	0.35
C20:5*n*−3	14.96	0.06	0.13	0.35	0.47	—	4.54	2.37	2.52	2.89	2.97	2.60	2.56	2.48	2.57
C22:6*n*−3	12.38	0.02	0.34	0.30	0.40	—	4.00	2.15	3.18	3.19	3.24	2.75	3.12	2.95	3.21
∑*n*−3 HUFA^c^	27.43	0.08	0.47	0.65	0.87	7.25^d^	8.59	4.57	5.74	6.13	6.26	5.40	5.73	5.48	5.83
Unknown	2.39	0.99	0.56	2.53	2.42	—	4.21	3.14	3.1	4.02	3.92	4.09	3.9	3.68	3.49

Abbreviations: CBM, chicken by-product meal; CGM, corn gluten meal; CPC, corn protein concentrate; MM, meat meal

^a^∑SFA: total saturated fatty acids.

^b^∑MUFA: total monounsaturated fatty acids.

^c^∑*n*−3 HUFA: total *n*−3 highly unsaturated fatty acids.

^d^Data were obtained from Lee et al. [51]'s study.

**Table 4:** Survival (%), weight gain (g/fish), and specific growth rate (SGR) of rockfish fed the experimental diets for 12 weeks.

Experimental diets	Initial weight (g/fish)	Final weight (g/fish)	Survival (%)	Weight gain (g/fish)	SGR^1^ (%/day)
Con	2.5 ± 0.04	36.1 ± 1.04	100.0 ± 0.00	33.7 ± 1.13^a^	3.20 ± 0.034^a^
MM	2.5 ± 0.03	27.8 ± 2.01	100.0 ± 0.00	25.4 ± 2.02^b^	2.87 ± 0.096^b^
CBM	2.5 ± 0.04	27.1 ± 0.15	100.0 ± 0.00	24.7 ± 0.14^b^	2.86 ± 0.019^b^
CGM	2.5 ± 0.02	25.1 ± 1.87	100.0 ± 0.00	22.6 ± 1.86^b^	2.76 ± 0.087^b^
CPC	2.5 ± 0.02	22.8 ± 0.71	100.0 ± 0.00	20.4 ± 0.69^b^	2.65 ± 0.027^b^
CMCG	2.5 ± 0.02	29.1 ± 1.48	100.0 ± 0.00	26.6 ± 1.46^b^	2.94 ± 0.048^ab^
CMCP	2.5 ± 0.00	24.1 ± 1.39	100.0 ± 0.00	21.6 ± 1.39^b^	2.71 ± 0.069^b^
CCCG	2.5 ± 0.02	24.3 ± 1.35	100.0 ± 0.00	21.8 ± 1.35^b^	2.72 ± 0.072^b^
CCCP	2.5 ± 0.02	25.0 ± 1.19	100.0 ± 0.00	22.5 ± 1.21^b^	2.75 ± 0.065^b^
*p*-Value	—	—	—	*p* < 0.0001	*p* < 0.001

*Note:* Values (means of triplicate ± SE) in the same column sharing the same superscript letter are not significantly different (*p*  > 0.05).

Abbreviations: CBM, chicken by-product meal; CGM, corn gluten meal; CPC, corn protein concentrate; MM, meat meal.

^1^Specific growth rate (SGR, %/day) = [Ln final body weight of fish (g) − Ln initial body weight of fish (g)] × 100/days of feeding.

**Table 5:** Feed consumption (g/fish), feed efficiency (FE), protein efficiency ratio (PER), protein retention (PR), condition factor (CF), viscerosomatic index (VSI), and hepatosomatic index (HSI) of rockfish fed the experimental diets for 12 weeks.

Experimental diets	Feed consumption (g/fish)	FE^1^	PER^2^	PR^3^ (%)	CF^4^ (g/cm^3^)	VSI^5^ (%)	HSI^6^ (%)
Con	32.4 ± 0.59	1.04 ± 0.018^a^	2.00 ± 0.035^a^	31.14 ± 1.406^a^	1.79 ± 0.046	10.91 ± 0.507	3.10 ± 0.202
MM	30.7 ± 0.21	0.83 ± 0.062^b^	1.56 ± 0.118^b^	24.63 ± 1.543^abc^	1.84 ± 0.032	10.40 ± 0.459	3.11 ± 0.175
CBM	30.8 ± 0.14	0.80 ± 0.008^b^	1.53 ± 0.014^b^	22.67 ± 1.386^bc^	1.80 ± 0.060	10.08 ± 0.475	2.96 ± 0.299
CGM	31.0 ± 0.73	0.73 ± 0.058^b^	1.38 ± 0.110^b^	21.29 ± 1.650^bc^	1.76 ± 0.022	10.72 ± 0.590	3.36 ± 0.244
CPC	29.8 ± 0.55	0.68 ± 0.014^b^	1.31 ± 0.026^b^	20.20 ± 1.506^bc^	1.71 ± 0.073	10.22 ± 0.423	3.04 ± 0.149
CMCG	30.6 ± 0.54	0.87 ± 0.036^ab^	1.66 ± 0.069^ab^	26.81 ± 1.422^ab^	1.82 ± 0043	10.53 ± 0.328	3.56 ± 0.155
CMCP	31.2 ± 0.69	0.70 ± 0.056^b^	1.33 ± 0.108^b^	18.87 ± 2.325^c^	1.76 ± 0.026	9.64 ± 0.333	3.13 ± 0.151
CCCG	30.4 ± 0.28	0.72 ± 0.050^b^	1.36 ± 0.095^b^	19.38 ± 1.529^bc^	1.80 ± 0.018	10.29 ± 0.283	3.05 ± 0.137
CCCP	30.2 ± 0.57	0.74 ± 0.027^b^	1.42 ± 0.051^b^	21.53 ± 0.411^bc^	1.75 ± 0.029	9.60 ± 0.808	3.13 ± 0.093
*p*-Value	*p* > 0.07	*p* < 0.0001	*p* < 0.0001	*p* < 0.0001	*p* > 0.5	*p* > 0.5	*p* > 0.4

*Note:* Values (means of triplicate ± SE) in the same column sharing the same superscript letter are not significantly different (*p* > 0.05).

Abbreviations: CBM, chicken by-product meal; CGM, corn gluten meal; CPC, corn protein concentrate; MM, meat meal.

^1^FE = weight gain of fish/feed consumption.

^2^PER = weight gain of fish/protein consumption.

^3^PR (%)  = protein gain of fish × 100/protein consumption.

^4^CF (g/cm^3^) = fish weight (g) × 100/total length (cm)^3^.

^5^VSI (%) = viscera weight × 100/fish weight.

^6^HSI (%) = liver weight × 100/fish weight.

**Table 6:** Proximate composition (% of wet weight) of the whole body of rockfish fed the experimental diets for 12 weeks.

Experimental diets	Moisture	Crude protein	Crude lipid	Ash
Con	69.8 ± 0.74	15.6 ± 0.82	8.4 ± 0.79	4.2 ± 0.19
MM	69.8 ± 0.74	15.8 ± 0.21	7.5 ± 1.04	4.9 ± 0.40
CBM	71.8 ± 1.80	14.9 ± 0.92	5.9 ± 0.66	4.4 ± 0.17
CGM	70.3 ± 0.73	15.5 ± 0.43	7.8 ± 0.89	4.2 ± 0.61
CPC	72.3 ± 2.25	15.4 ± 0.86	6.7 ± 0.16	4.4 ± 0.02
CMCG	71.7 ± 1.65	16.1 ± 0.45	7.3 ± 0.73	4.5 ± 0.01
CMCP	72.7 ± 1.51	14.2 ± 0.62	7.5 ± 1.24	4.1 ± 0.13
CCCG	74.5 ± 0.49	14.4 ± 0.12	6.0 ± 0.65	3.9 ± 0.23
CCCP	72.5 ± 2.28	15.2 ± 0.31	6.6 ± 0.83	4.2 ± 0.26
*p*-Value	*p* > 0.4	*p* > 0.5	*p* > 0.4	*p* > 0.4

Abbreviations: CBM, chicken by-product meal; CGM, corn gluten meal; CPC, corn protein concentrate; MM, meat meal.

**Table 7:** Blood chemistry of rockfish fed the experimental diets for 12 weeks.

	Experimental diets									
	Con	MM	CBM	CGM	CPC	CMCG	CMCP	CCCG	CCCP	*p*-Value
Plasma parameters										
AST (IU/L)	116.0 ± 4.19	116.0 ± 8.89	123.3 ± 10.27	120.3 ± 2.74	117.7 ± 9.98	156.7 ± 2.40	155.3 ± 17.75	106.0 ± 3.28	137.3 ± 4.76	*p* > 0.4
ALT (IU/L)	13.7 ± 1.09	18.0 ± 0.76	20.7 ± 2.09	23.3 ± 1.17	19.7 ± 1.69	25.0 ± 2.52	24.7 ± 3.53	22.0 ± 1.15	19.7 ± 0.67	*p* > 0.5
ALP (IU/L)	287.7 ± 55.08	392.3 ± 22.08	485.7 ± 22.66	526.3 ± 34.40	475.7 ± 53.43	420.3 ± 17.59	218.7 ± 58.09	449.3 ± 26.83	212.3 ± 22.66	*p* > 0.5
T-BIL (mg/dL)	1.9 ± 0.15	2.7 ± 0.23	3.0 ± 0.25	2.8 ± 0.06	3.0 ± 0.39	2.2 ± 0.23	2.4 ± 0.25	1.8 ± 0.18	1.7 ± 0.09	*p* > 0.2
T-CHO (mg/dL)	212.7 ± 1.86	220.0 ± 4.09	225.0 ± 5.13	211.3 ± 2.62	238.0 ± 4.65	238.7 ± 4.97	240.0 ± 11.32	251.7 ± 14.24	218.0 ± 4.01	*p* > 0.4
TG (mg/dL)	309.3 ± 13.92	392.3 ± 11.40	455.0 ± 11.81	397.7 ± 17.85	431.7 ± 26.13	455.7 ± 21.55	472.0 ± 44.53	383.7 ± 25.44	364.3 ± 35.11	*p* > 0.4
TP (g/dL)	5.0 ± 0.17	5.3 ± 0.26	6.0 ± 0.27	5.3 ± 0.09	5.3 ± 0.38	5.0 ± 0.39	4.8 ± 0.21	5.4 ± 0.32	4.8 ± 0.25	*p* > 0.8
ALB (g/dL)	1.5 ± 0.18	1.9 ± 0.09	2.1 ± 0.24	2.1 ± 0.14	1.9 ± 0.19	1.8 ± 0.09	1.6 ± 0.18	1.9 ± 0.06	1.1 ± 0.06	*p* > 0.3
Serum parameters										
Lysozyme activity (U/mL)	756.6 ± 52.05	746.8 ± 26.18	749.0 ± 19.23	753.9 ± 24.80	751.3 ± 15.55	752.2 ± 15.41	755.0 ± 43.11	753.3 ± 19.22	750.6 ± 26.25	*p* > 0.9
SOD (ng/mL)	3.8 ± 0.06	4.0 ± 0.06	4.1 ± 0.13	4.0 ± 0.14	3.8 ± 0.14	4.1 ± 0.10	3.7 ± 0.10	3.9 ± 0.00	4.0 ± 0.11	*p* > 0.6

Abbreviations: ALB, albumin; ALP, alkaline phosphatase; ALT, alanine aminotransferase; AST, aspartate aminotransferase; CBM, chicken by-product meal; CGM, corn gluten meal; CPC, corn protein concentrate; MM, meat meal; SOD, superoxide dismutase; T-BIL, total bilirubin; T-CHO, total cholesterol; TG, triglyceride; TP, total protein.

**Table 8:** Amino acid (% of wet weight) compositions of the whole body of rockfish fed the experimental diets for 12 weeks.

	Experimental diets									
	Con	MM	CBM	CGM	CPC	CMCG	CMCP	CCCG	CCCP	*p*-Value
Essential amino acid (%)										
Arginine	1.22 ± 0.038	1.18 ± 0.015	1.20 ± 0.029	1.23 ± 0.006	1.15 ± 0.049	1.21 ± 0.006	1.19 ± 0.006	1.13 ± 0.055	1.23 ± 0.009	*p* > 0.3
Histidine	0.52 ± 0.058	0.48 ± 0.055	0.48 ± 0.049	0.51 ± 0.020	0.44 ± 0.006	0.47 ± 0.017	0.48 ± 0.020	0.47 ± 0.009	0.50 ± 0.107	*p* > 0.9
Isoleucine	1.04 ± 0.020	0.96 ± 0.196	0.95 ± 0.144	0.94 ± 0.015	0.83 ± 0.015	0.88 ± 0.012	0.86 ± 0.012	0.83 ± 0.026	0.85 ± 0.026	*p* > 0.6
Leucine	1.72 ± 0.035	1.61 ± 0.043	1.66 ± 0.127	1.69 ± 0.020	1.58 ± 0.055	1.64 ± 0.006	1.61 ± 0.015	1.60 ± 0.043	1.71 ± 0.006	*p* > 0.5
Lysine	2.04 ± 0.015	1.90 ± 0.055	1.95 ± 0.072	1.98 ± 0.153	1.77 ± 0.268	1.92 ± 0.026	1.88 ± 0.043	1.88 ± 0.009	1.99 ± 0.020	*p* > 0.8
Methionine	0.74 ± 0.015	0.69 ± 0.015	0.73 ± 0.121	0.70 ± 0.289	0.74 ± 0.017	0.70 ± 0.058	0.64 ± 0.032	0.66 ± 0.026	0.66 ± 0.015	*p* > 0.9
Phenylalanine	0.92 ± 0.043	0.88 ± 0.015	0.86 ± 0.066	0.87 ± 0.055	0.79 ± 0.032	0.81 ± 0.012	0.82 ± 0.006	0.80 ± 0.065	0.86 ± 0.006	*p* > 0.4
Threonine	0.87 ± 0.130	0.89 ± 0.118	0.94 ± 0.090	0.98 ± 0.035	0.87 ± 0.069	0.95 ± 0.087	0.94 ± 0.090	0.95 ± 0.087	1.02 ± 0.046	*p* > 0.9
Tryptophan	0.16 ± 0.032	0.15 ± 0.043	0.15 ± 0.078	0.15 ± 0.032	0.15 ± 0.023	0.15 ± 0.020	0.13 ± 0.009	0.14 ± 0.006	0.16 ± 0.009	*p* > 0.9
Valine	1.10 ± 0.026	1.00 ± 0.113	0.99 ± 0.179	0.99 ± 0.046	0.87 ± 0.035	0.93 ± 0.015	0.91 ± 0.006	0.88 ± 0.012	0.90 ± 0.052	*p* > 0.4
Non-essential amino acid (%)										
Alanine	1.35 ± 0.020	1.28 ± 0.219	1.29 ± 0.038	1.32 ± 0.061	1.19 ± 0.006	1.28 ± 0.006	1.28 ± 0.015	1.27 ± 0.032	1.36 ± 0.015	*p* > 0.9
Aspartic acid	2.22 ± 0.081	2.08 ± 0.300	2.12 ± 0.185	2.17 ± 0.072	1.94 ± 0.017	2.10 ± 0.115	2.08 ± 0.049	2.09 ± 0.020	2.24 ± 0.020	*p* > 0.8
Cysteine	0.36 ± 0.049	0.34 ± 0.020	0.34 ± 0.139	0.34 ± 0.055	0.33 ± 0.015	0.30 ± 0.009	0.28 ± 0.040	0.40 ± 0.058	0.31 ± 0.006	*p* > 0.9
Glutamic acid	3.19 ± 0.121	2.96 ± 0.179	3.04 ± 0.127	2.98 ± 0.142	2.77 ± 0.361	3.08 ± 0.015	2.98 ± 0.020	3.01 ± 0.023	3.19 ± 0.026	*p* > 0.7
Glycine	1.16 ± 0.107	1.16 ± 0.127	1.10 ± 0.404	1.11 ± 0.015	0.99 ± 0.046	1.10 ± 0.115	1.12 ± 0.020	1.09 ± 0.015	1.12 ± 0.009	*p* > 0.9
Proline	0.72 ± 0.038	0.73 ± 0.040	0.71 ± 0.038	0.78 ± 0.015	0.66 ± 0.078	0.74 ± 0.006	0.74 ± 0.006	0.74 ± 0.015	0.76 ± 0.006	*p* > 0.5
Serine	0.70 ± 0.039	0.76 ± 0.032	0.83 ± 0.072	0.87 ± 0.101	0.79 ± 0.052	0.88 ± 0.072	0.87 ± 0.066	0.88 ± 0.072	0.94 ± 0.113	*p* > 0.4
Tyrosine	0.45 ± 0.101	0.53 ± 0.064	0.62 ± 0.009	0.64 ± 0.090	0.59 ± 0.035	0.66 ± 0.092	0.65 ± 0.081	0.64 ± 0.038	0.70 ± 0.115	*p* > 0.5

Abbreviations: CBM, chicken by-product meal; CGM, corn gluten meal; CPC, corn protein concentrate; MM, meat meal.

**Table 9:** Fatty acids (% of total fatty acids) profiles of rockfish fed the experimental diets for 12 weeks.

	Experimental diets									
	Con	MM	CBM	CGM	CPC	CMCG	CMCP	CCCG	CCCP	*p*-Value
C12:0	0.08 ± 0.016	0.04 ± 0.018	0.15 ± 0.053	0.04 ± 0.018	0.04 ± 0.018	0.15 ± 0.063	0.04 ± 0.018	0.05 ± 0.023	0.05 ± 0.020	*p* > 0.2
C14:0	2.38 ± 0.238	1.98 ± 0.238	1.96 ± 0.038	1.54 ± 0.518	1.62 ± 0.190	1.70 ± 0.250	1.70 ± 0.200	1.58 ± 0.183	1.75 ± 0.478	*p* > 0.7
C16:0	16.78 ± 0.060	16.46 ± 0.108	17.05 ± 0.248	14.52 ± 0.813	14.47 ± 1.513	15.03 ± 0.688	15.43 ± 0.663	15.92 ± 0.588	16.20 ± 0.650	*p* > 0.3
C18:0	4.22 ± 0.213	4.98 ± 0.388	5.19 ± 0.543	4.51 ± 1.003	4.45 ± 0.825	4.54 ± 0.205	4.69 ± 0.110	5.12 ± 0.338	4.83 ± 0.055	*p* > 0.9
C20:0	0.14 ± 0.023	0.13 ± 0.058	0.14 ± 0.033	0.12 ± 0.043	0.14 ± 0.043	0.13 ± 0.038	0.14 ± 0.045	0.14 ± 0.013	0.14 ± 0.068	*p* > 0.9
C24:0	0.75 ± 0.085	0.61 ± 0.048	0.63 ± 0.175	0.60 ± 0.298	0.67 ± 0.128	0.65 ± 0.200	0.60 ± 0.290	0.62 ± 0.183	0.61 ± 0.100	*p* > 0.9
∑SFA^1^	24.34 ± 0.220	24.20 ± 0.401	25.11 ± 0.280	21.33 ± 2.629	21.39 ± 2.702	22.19 ± 0.629	22.60 ± 0.144	23.43 ± 0.658	23.59 ± 1.149	*p* > 0.4
C14:1*n*−5	0.06 ± 0.013	0.07 ± 0.028	0.07 ± 0.030	0.06 ± 0.023	0.04 ± 0.018	0.05 ± 0.020	0.05 ± 0.020	0.10 ± 0.038	0.10 ± 0.025	*p* > 0.8
C15:1*n−*7	0.04 ± 0.015	0.04 ± 0.018	0.05 ± 0.013	0.04 ± 0.018	0.05 ± 0.023	0.04 ± 0.018	0.03 ± 0.010	0.05 ± 0.023	0.04 ± 0.015	*p* > 0.9
C16:1*n*−7	4.69 ± 0.598	3.86 ± 0.380	3.71 ± 0.798	2.98 ± 0.423	2.99 ± 0.905	3.20 ± 0.700	3.32 ± 0.760	3.37 ± 0.638	3.82 ± 0.338	*p* > 0.8
C17:1*n*−7	0.31 ± 0.135	0.29 ± 0.055	0.31 ± 0.098	0.25 ± 0.068	0.15 ± 0.025	0.26 ± 0.045	0.26 ± 0.073	0.18 ± 0.058	0.27 ± 0.083	*p* > 0.9
C18:1*n*−9	30.94 ± 0.569	32.29 ± 1.000	31.67 ± 1.094	32.60 ± 0.900	30.96 ± 0.639	31.64 ± 0.338	32.13 ± 0.925	31.40 ± 1.075	33.19 ± 0.391	*p* > 0.7
C20:1*n*−9	1.34 ± 0.070	1.18 ± 0.035	1.16 ± 0.080	1.11 ± 0.043	1.18 ± 0.083	1.14 ± 0.033	1.17 ± 0.030	1.11 ± 0.155	1.13 ± 0.060	*p* > 0.7
C22:1*n*−9	1.13 ± 0.090	0.77 ± 0.183	0.75 ± 0.025	0.74 ± 0.113	0.78 ± 0.323	0.74 ± 0.220	0.73 ± 0.210	0.70 ± 0.300	0.71 ± 0.095	*p* > 0.9
∑MUFA^2^	38.52 ± 1.117	38.49 ± 0.713	37.71 ± 1.798	37.78 ± 0.704	36.16 ± 1.456	37.07 ± 0.802	37.69 ± 0.157	36.91 ± 0.930	38.83 ± 0.671	*p* > 0.6
C18:2*n*−6	22.11 ± 2.257	23.82 ± 1.328	23.63 ± 2.785	27.95 ± 0.775	26.74 ± 1.175	26.51 ± 0.945	26.67 ± 0.740	26.12 ± 0.539	24.56 ± 0.532	*p* > 0.3
C18:3*n*−3	2.99 ± 0.108	3.00 ± 0.450	2.66 ± 0.273	3.33 ± 0.148	3.39 ± 0.138	3.29 ± 0.655	3.23 ± 0.188	2.86 ± 0.048	2.75 ± 0.098	*p* > 0.7
C18:3*n*−6	0.21 ± 0.068	0.20 ± 0.038	0.20 ± 0.090	0.23 ± 0.095	0.23 ± 0.080	0.21 ± 0.088	0.21 ± 0.043	0.21 ± 0.088	0.23 ± 0.050	*p* > 0.9
C20:2*n*−6	0.29 ± 0.063	0.31 ± 0.100	0.31 ± 0.093	0.39 ± 0.118	0.40 ± 0.158	0.36 ± 0.073	0.35 ± 0.068	0.32 ± 0.075	0.32 ± 0.068	*p* > 0.9
C20:3*n*−3	0.08 ± 0.013	0.08 ± 0.033	0.08 ± 0.015	0.08 ± 0.028	0.10 ± 0.013	0.08 ± 0.030	0.08 ± 0.030	0.07 ± 0.025	0.07 ± 0.025	*p* > 0.9
C20:3*n*−6	0.12 ± 0.023	0.13 ± 0.038	0.14 ± 0.055	0.14 ± 0.055	0.13 ± 0.028	0.13 ± 0.048	0.13 ± 0.050	0.13 ± 0.028	0.14 ± 0.018	*p* > 0.9
C20:4*n*−6	0.49 ± 0.120	0.47 ± 0.063	0.52 ± 0.103	0.40 ± 0.150	0.47 ± 0.118	0.46 ± 0.170	0.43 ± 0.125	0.51 ± 0.095	0.47 ± 0.058	*p* > 0.9
C20:5*n*−3	4.11 ± 0.015	3.06 ± 0.220	3.17 ± 0.163	2.74 ± 0.470	3.28 ± 0.013	3.04 ± 0.380	2.90 ± 0.875	3.03 ± 0.238	3.07 ± 0.488	*p* > 0.7
C22:6*n*−3	4.37 ± 0.068	3.48 ± 0.348	3.82 ± 0.288	3.28 ± 0.788	4.03 ± 0.013	3.72 ± 0.545	3.39 ± 0.035	3.81 ± 0.705	3.51 ± 0.353	*p* > 0.8
∑*n*−3 HUFA^3^	8.55 ± 0.110^a^	6.63 ± 0.110^b^	7.08 ± 0.127^b^	6.09 ± 0.398^b^	7.40 ± 0.012^b^	6.84 ± 0.225^b^	6.37 ± 1.005^b^	6.91 ± 0.511^b^	6.65 ± 0.185^b^	*p* < 0.02
Unknown	2.38 ± 1.720	2.76 ± 2.094	2.65 ± 2.010	2.37 ± 1.207	3.69 ± 2.748	2.95 ± 2.217	2.32 ± 1.484	2.60 ± 1.845	2.47 ± 1.853	*p* > 0.9

*Note:* Values (means of triplicate ± SE) in the same row sharing the same superscript letter are not significantly different (*p*  > 0.05).

Abbreviations: CBM, chicken by-product meal; CGM, corn gluten meal; CPC, corn protein concentrate; HUFA, highly unsaturated fatty acid; MM, meat meal; MUFA, monounsaturated fatty acid.

^1^∑SFA: total saturated fatty acids.

^2^∑MUFA: total monounsaturated fatty acids.

^3^∑*n*-3 HUFA: total *n*-3 highly unsaturated fatty acids.

## Data Availability

Data available on request from the authors.
